# Algal Metabolites Can Be an Immune Booster against COVID-19 Pandemic

**DOI:** 10.3390/antiox11030452

**Published:** 2022-02-24

**Authors:** Ajay Kumar, Rahul Prasad Singh, Indrajeet Kumar, Priya Yadav, Sandeep Kumar Singh, Kaushalendra, Prashant Kumar Singh, Rajan Kumar Gupta, Shiv Mohan Singh, Mahipal Singh Kesawat, Ganesh Dattatraya Saratale, Sang-Min Chung, Manu Kumar

**Affiliations:** 1 Volcani Center, Department of Postharvest Science, Agriculture Research Organization, Rishon LeZion 7505101, Israel; ajaykumar_bhu@yahoo.com; 2 Centre of Advanced Study in Botany, Institute of Science, Banaras Hindu University, Varanasi 221005, India; rah.singhbhu@gmail.com (R.P.S.); indrajeetk991@gmail.com (I.K.); priya02061995@gmail.com (P.Y.); rajankgupta1@rediffmail.com (R.K.G.); drshivmohansingh@gmail.com (S.M.S.); 3 Division of Microbiology, Indian Agricultural Research Institute, Pusa, New Delhi 110012, India; sandeepksingh015@gmail.com; 4 Department of Zoology, Pachhunga University College Campus, Mizoram University (A Central University), Aizawl 796001, India; kaushalpuc@gmail.com; 5 Department of Biotechnology, Pachhunga University College Campus, Mizoram University (A Central University), Aizawl 796001, India; prashantbotbhu@gmail.com; 6 Department of Genetics and Plant Breeding, Faculty of Agriculture, Sri Sri University, Cuttack 754006, India; mahipal.s@srisriuniversity.edu.in; 7 Department of Food Science and Biotechnology, Dongguk University, Seoul 10326, Korea; gdsaratale@gmail.com; 8 Department of Life Science, College of Life Science and Biotechnology, Dongguk University, Seoul 10326, Korea; smchung@dongguk.edu

**Keywords:** algae, antioxidants, antiviral, COVID-19, metabolites, SARS-CoV-2

## Abstract

The world has faced the challenges of Severe Acute Respiratory Syndrome Coronavirus 2 (SARS-CoV-2) for the last two years, first diagnosed at the end of 2019 in Wuhan and widely distributed worldwide. As a result, the WHO has proclaimed the illness brought on by this virus to be a global pandemic. To combat COVID-19, researcher communities continuously develop and implement rapid diagnoses, safe and effective vaccinations and other alternative therapeutic procedures. However, synthetic drug-related side effects and high costs have piqued scientists’ interest in natural product-based therapies and medicines. In this regard, antiviral substances derived from natural resources and some medicines have seen a boom in popularity. For instance, algae are a rich source of compounds such as lectins and sulfated polysaccharides, which have potent antiviral and immunity-boosting properties. Moreover, Algae-derived compounds or metabolites can be used as antibodies and vaccine raw materials against COVID-19. Furthermore, some algal species can boost immunity, reduce viral activity in humans and be recommended for usage as a COVID-19 preventative measure. However, this field of study is still in its early stages of development. Therefore, this review addresses critical characteristics of algal metabolites, their antioxidant potential and therapeutic potential in COVID-19.

## 1. Introduction

Since ancient times, plants/plant parts/natural products (extracts and/or metabolites) have been directly or indirectly utilized in allopathic or Ayurveda to cure various human diseases [1,2,3,4]. According to published records, it has been estimated that approximately 40–50% of total known plant species have been used for medicinal purposes [5,6]. Furthermore, more than 25% of modern medicine and nearly 80% of the global population depend upon plant metabolites for primary health treatments [7,8,9]. Algae and algae-derived products are rich sources of natural products or metabolites synthesized during metabolism [10]. These metabolites possess various pharmacological activities, including antibacterial, analgesic, antiviral, etc. In addition, the structural diversity of compounds or constituents such as alkaloids, terpenes, polyphenols, sterols, lactones offers an opportunity for bioactivity and drug design [11,12].

An unexpected outbreak of viral illnesses has wreaked havoc on human health, posing a severe health hazard and economic damage. In the recent past, some of the spontaneous outbreaks, such as SARS (severe acute respiratory syndrome) in 2002–2003, H_1_N_1_ influenza in 2009 and MERS (Middle East respiratory disease) in 2012, severely affected human life [13]. The COVID-19 (Coronavirus Infectious Disease 2019) pandemic began in late December 2019 with an unknown etiology, which was later determined to be caused by a novel coronavirus variant identified as 2019-nCoV/SARS-CoV-2 [14]. The virulence of COVID-19 is so high that, in a few months, it spread throughout the world and appeared as a global pandemic. However, scientific communities have made significant progress in the last two years in developing vaccines, which do not entirely control the virus infection but evoke the immune system to act against the disease. However, the latest mutant strains still pose a severe threat and need additional precautions and alternatives to combat the COVID-19 pandemic or enhance the immune response. In this regard, utilizing natural products or plant metabolites appears suitable to boost the immune system against this pandemic.

Algae are among the most widespread aquatic, photosynthetic organisms present in both freshwater and marine water [15,16]. Algae are considered an excellent source of secondary metabolites or bioactive compounds [17,18,19]. The cultivation system, growth conditions and growth phases are some prime factors that limit the rate and amount of metabolite production [20,21,22]. Similar to other plant-derived molecules, algal metabolites constitute various compound classes, such as polyphenols, lipids, phytols, terpenes, pigments, sterols, free fatty acids, pigments, vitamins, amino acids, peptides, polysaccharides, chitooligosaccharide and halogenated compounds. These compounds possess a broad range of pharmacological activities [23]. Various authors reported algae-derived metabolites and their significant effect in treating human ailments [24]. These metabolites have also been investigated to enrich pharmaceutical properties to treat various human disorders. For instance, calcium-rich spirulina derived from *Spirulina platensis* possesses antiviral activity against many human diseases [25]. Similarly, nostoflan, derived from *Nostoc flagelliforme,* exhibited antiviral activity against the influenza A virus [26]. Cyanovirin-N (CV-N) is a protein derived from *Nostoc ellipsosporum* which also displayed antiviral action against severe viral diseases, such as human immunodeficiency and simian immunodeficiency [27,28,29].

However, more efforts are being undertaken to discover therapy methods that effectively combat the current COVID-19 pandemic triggered by Coronavirus Severe Acute Respiratory Syndrome Coronavirus 2 (SARS-CoV-2). Therefore, antiviral compounds in algae must be investigated to identify a viable treatment resource for SARS-CoV-2 [30]. The SARS-CoV-2 genome encodes a variety of structural (spike glycoprotein), non-structural (helicase, 3-chymotrypsin-like, papain-like protease, protease and RNA-dependent RNA polymerase) and auxiliary proteins [31]. It is thought that the spike glycoprotein is essential when viruses and host cell receptors interact. Therefore, much recent research has focused on this structural protein, since it is crucial for virus entrance into host cells [32]. Our review discusses the potential antiviral activity of algal metabolites and possible therapeutics against SARS-CoV-2 treatment.

## 2. Antiviral Metabolites Generated from Algae against SARS-CoV-2

The antiviral activity of many marine algal species and their extracts and metabolites have been reviewed established as antiviral agents against many viruses [33,34]. *Spirulina* is a dietary supplement with many essential fatty acids, phenolic acids, vitamin B12 and sulfated polysaccharides. It has antiviral properties against pseudo-type coronaviruses because it binds to the 36 spikes of the S1 domain and prevents spikes from interacting with their receptor [35]. Therefore, red algal species *Porphyridium* sulfated polysaccharides are advocated to serve as promising antiviral medicines that could be utilized to coat sanitary products to prevent COVID-19 [36]. In addition, natural astaxanthin (nASX) extracted from microalgal species (*Haematococcus Pluvialis*) served as an adjuvant in combination with primary COVID-19 drugs by enhancing their immunity and shortening the period of patient recovery [37]. Similarly, an unusual diterpene derived from the *Halimeda tuna* possesses antiviral activity against murine coronavirus [38]. Red algae-derived Griffithsin has antiviral properties against MERS-CoV and SARS-CoV [39,40]. In vitro, Griffithsin inhibits a wide range of CoVs in SARS-CoV-infected mice, including HCoV-OC43, HCV-229E and HCV-NL63 [41], because it blocks virus entry and integrase activity, as well as protease and reverse transcriptase activities [42]. Other polysaccharides, such as ulvans (produced from green algae) and fucoidans (derived from brown algae), were also being investigated as potential SARS-CoV-2 therapeutic agents [43]. *Arthrospira*-derived phycoerythrobilin, phycocyanobilin and folic acid compete with SARS-CoV-2 for binding [44]. However, an *in-silico* experiment was conducted to screen therapeutic SARS-CoV targets using algae-derived compounds obtained from *Gracilaria corticata*, *Laurencia papillosa and Grateloupia filicina,* which have exhibited antiviral activities against SARS-CoV-2 [45].

Phlorotannins, alginates, luminaries, fucoidans, polyphenols, carotenoids, carrageenans and fatty acids are marine algae-derived bioactive chemicals that enhance the human gut microbiota and sustain host health by enhancing epithelial barrier integrity, immune system function and metabolism modulation [46,47]. Indeed, improving diets, mainly proteins, vitamins, minerals and a fiber-rich food, can help boost the immune system and is considered an effective strategy for combating COVID-19 [48]. The food products derived from marine algal seaweed are rich in proteins and vitamins, making them suitable for supplementation in the context of hypovitaminosis risk factors. Carotenoids, phytosterols, fatty acids and vitamins derived from various microalgal species have also been shown to have immunological activity [49]. Consumption of the green alga *Chlamydomonas reinhardtii* has been proven to relieve human gastrointestinal disorders such as Irritable Bowel Syndrome (IBS), linked to gas, diarrhea and bloating problems [50]. In the ileum of elderly mice, *spirulina* modified many immunological activities in the stomach and upregulated the expression of the 2 and 4 toll-like receptors (TLR2 and TLR4) [51]. Modified pectin produced from *Spirulina maxima* altered gut microbiota and induced immunological responses in mice [52]. Brown seaweed *Ascophyllum nodosum*-derived sulfated polysaccharides have been shown to regulate gut microbiota’s functional and structural aspect by modifying the abundance of beneficial bacteria as a functional food [53].

The previous study showed the effectiveness of algal polysaccharides in modifying the gut microbiota and improving gut health [54]. Furthermore, *Sargassum muticum* and *Osmundea* extracts were also exploited as novel functional foods for the human gut microbiome [55]. As a result, optimizing the gut microbiome and its metabolites, as well as creating a tailored diet, can be an effective strategy in preventing and treating COVID-19 [56]. A list of compounds derived from algae that may be effective against COVID-19 and other viral diseases is shown in Table 1.

### 2.1. Phycocyanobilins Antiviral Chromospheres against SARS-CoV-2

**Figure 1:**
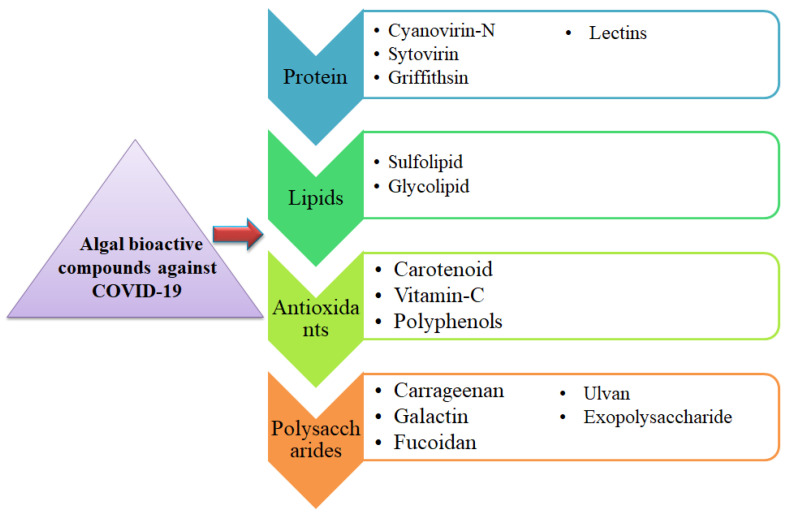
Bioactive metabolites extracted from algae and their possible approach to treating or preventing COVID-19.

Phycocyanobilins (PCBs) are blue phycobilins, which are tetrapyrrole chromophores found in cyanobacteria and rhodophytes (Figure 1) [65]. The antioxidant, antiviral and NADPH-oxidase inhibitory activities of these light-capturing pigments are intensively investigated [66,67,68]. Potential SARS-CoV-2 infection inhibitors are made from PCBs (source: *Spirulina* sp.) [69]. The bioactive compounds were screened in silico to utilize anti-SARS-CoV-2 activity through the COVID-19 Docking Server. Phycocyanobilin has been found to have a strong affinity for binding two possible targets, RNA-dependent RNA polymerase (RdRp) and the Main protease (M^pro^). Polyprotein digestion is carried out by the primary protease (translated from SARS-CoV-2 RNA), whereas the polymerase is responsible for viral RNA replication. The study found PCB binding target enzymes with substantial antiviral potential. However, as it is already known, in vitro or in vivo research is required to back up the docking results and discover PCBs’ true potential as a COVID-19 treatment [69].

In addition, pure allophycocyanin from *Spirulina platensis* has considerable antiviral action against enteroviruses [70]. Similarly, in an in silico research study, PCB produced by *Arthrospira* sp. was discovered to be an effective antiviral against SARS-CoV-2 [71]. In *Arthrospira* sp., PCB interactions with the SARS-CoV-2 spike glycoproteins receptor-binding domain (RBD) were studied. The PCB/Spike RBD complex was found to have five Van der Waals contacts, such as ASN501, GLN493, LEU492, TYR453 and ARG403. The residues TYR505, PHE497, TYR495 and TYR449 shared PCB and the spike RBD to form five p-alkyl linkages and TYR449 formed a hydrogen bond. SER494, GLY496 and GLN498 were the other residues that included a hydrogen bond, with GLY496 being linked to PCB via a hydrogen bond with a π-donor. Finally, the binding energy from −7.25 to −9.355 kcal·mol^−1^ was competitive, indicating that PCB might be used against viruses [71].

In addition, a recent study suggested that cyanobacteria that contain phycocyanobilin, such as *Spirulina* sp., could be used to treat infections that are caused by RNA viruses [72]. In animal research experiments, cold-water phycocyanin-rich extracts of *spirulina* sp. were given orally to influenza-infected mice, which resulted in a lower mortality rate. Furthermore, the cold-water extract was well tolerated even at high doses of 3000 mg/kg/day in animal models for 14 days [73]. Certain algal PCB chromophore has been shown to possess NAPDH oxidase inhibiting activity with significant antioxidant and anti-inflammatory effects. Hence, the ingestion of algal extracts enriched in PCB might enhance the type 1 interferon response in the context of RNA virus infection [72]. As a result, it is expected that PCB-producing microalgae would show significant efficacy against SARS-CoV-2 [74,75]. Furthermore, more studies and in vivo investigations are required to fully comprehend the unique bioactivity of PCBs to create therapeutic techniques against viruses that cause human disease, such as SARS-CoV-2.

### 2.2. Antiviral Therapies Based on Algal Glycan against SARS-CoV-2

Most algae are enriched with several pharmacologically active compounds with antiviral activities [76]. As a result, we postulated that antiviral medicines derived from cyanobacteria could be used to combat the virus that causes COVID-19 [77,78]. Glycoproteins are characterized by a broad spectrum of biological activities, including antiviral characteristics, shown in Table 2. The spike (S) glycoproteins interact with the glycans of the cell surface and make an initial connection with the glycoprotein of the host cell and virus envelope. The glycoprotein-based antiviral therapy is an emerging research paradigm [79,80].

The glycosylation of viral envelope proteins is essential for infectivity. In addition, it can influence immune recognition [81]. These glycoproteins are large, containing 23–38 N-linked glycan sites per protomer [82]. Therefore, another research project looked for 22 glycosylation sites where glycans were bonded to the SARS-CoV-2 spike [83]. Sixty-six glycosylation sites are present in the S-glycoprotein of SARS-CoV-2 [84]. The human serine protease (TMPRSS2) is responsible for priming SARS-S-glycoprotein and CoV-2 and angiotensin-converting enzyme 2 (ACE2) is a human enzyme that acts as a SARS-CoV-2 receptor entry [85].

Hemaglutinins (lectins) are carbohydrate-binding glycoproteins that reversibly bind to monosaccharides and oligosaccharides [86]. Red algal lectins were thrust into the spotlight when Griffithsin [87] from *Griffithsia* sp. was identified. Since then, it has been extensively researched for various usages [88]. It appears to have a strong affinity for residues of mannose found on viral glycoproteins. It has been shown, in a few trials, to have antiviral activity against HIV-1, Hepatitis C and SARS-CoV glycoproteins [89,90,91]. A current study looked at griffithsin anti-MERS-CoV efficacy and found that lectin limited virus entrance while causing minimal cellular damage [92]. Time course tests were used to test the inhibitory impact of Griffithsin on virus infection during the binding step. Thus, Griffithsin was shown to decrease MERS-CoV infectivity in vitro [39]. In addition, Griffithsin has antiviral action in vivo against Japanese encephalitis virus, herpes simplex virus 2 and human papillomavirus [92,93,94]. The rectal mucosal proteome and microbiota were not significantly impacted by 0.1% griffithsin gel. Furthermore, model mice infected with a high dosage of SARS-CoV were given a griffithsin dose of 10 mg/kg (b.w.)/day, resulting in 100 percent survival [95]. Based on its anti-SARS-CoV activity, Griffithsin could be studied as a treatment for SARS-CoV-2.

Similarly, a lectin that binds to D-mannose is named as GCL (Grateloupia chianggi lectin) in the red macroalgae *Grateloupia chianggi* [96]. GCL purity, molecular and functional characterization and antiviral properties against herpes simplex virus, influenza and HIV were the focus of the research study. GCL concentrations of 1–20 nM were needed to suppress HSV efficiently. As a result, it could be concluded that GCL could be applied in virology and biomedical research. The SARS-CoV-2 virus is linked to the influenza virus in that they are both enclosed RNA viruses [97,98]. The antiviral efficacy of GCL against the influenza virus suggests that it could be also tested against SARS-CoV-2.

### 2.3. Antiviral Therapies Based on Algal Sulfated Polysaccharides against SARS-CoV-2

A sulfated polymer derived from red algae, such as *Gigartina*, *Chondrus*, *Eucheuma* and *Hypnea*, prevented viruses from infecting host cells by preventing their binding or integration [110,111,112]. It prevented the dengue virus from replicating in the cells of mosquitoes and mammals. In addition, they work against a variety of human papillomavirus (HPV), a sexually transmitted strain that can cause genital warts and cervical cancer. Two fucoidans from *Sargassum henslowianum*, a brown macroalga, were purified and structurally characterized in a recent study [113]. SHAP-1 and SHAP-2 fucoidans were investigated for their ability to fight against HSV-1 and HSV-2 herpes simplex virus strains. The fucoidans were also involved in interrupting the adsorption of HSV to the host cell in the adsorption and penetration assays. As a result, fucoidans appear to be attractive candidates for inhibiting HSV-2 viruses and could be used successfully in various clinical settings.

Additionally, under established in vitro conditions, seaweed polysaccharides (SPs) produced from macroalgae *Cladosiphon okamuranus* and *Ulva clathrata* were found to have considerable antiviral activity against the virus that causes Newcastle disease [114]. Another research study has shown that the SPs derived from *Grateloupia filicina*, *Sargassum qingdaoense*, *Ulva pertusa* had antiviral properties. They performed in vitro and in vivo studies against the avian influenza virus [115]. *Monostroma nitidum,* green macroalgae, was also used to isolate a sulfated polysaccharide [116]. MWS (Water-soluble polysaccharide from *Monostroma nitidum*) was added to the substance isolated from *M. nitidum,* since it was discovered as a sulfated glucuronorhamnan that is water soluble. Several cytotoxicity and antiviral tests were used to evaluate its efficacy against EV71 (a human pathogenic enterovirus strain).

Carrageenans with a low molecular weight (3, 5 and 10 kDa) have been shown to have significant inhibitory effects against the influenza virus in in vivo investigations [110,117]. The usage of a nasal spray containing carrageenan (Iota-carrageenan), sometimes recognized as “super-shedders”, enhanced viral clearance, shortened duration and recurrences of the common cold and it is considered a successful treatment for the common cold [118,119]. Sulfated polysaccharides attach tightly to the SARS-S-protein and act as decoys in host tissues, preventing the S-protein from adhering to the heparin sulfate co-receptor and reducing viral infection [120]. On sanitary items, *Porphyridium* sulfated polysaccharides have been used as a covering material and in the manufacture of antiviral drugs [121]. *Porphyridium* exopolysaccharides, together with sulfated polysaccharides and carrageenan, prevented virus internalization or binding to host cells. As a result, they diminished COVID-19 growth and may be a viable antiviral drug against coronavirus-related respiratory infections [122].

**Figure 2:**
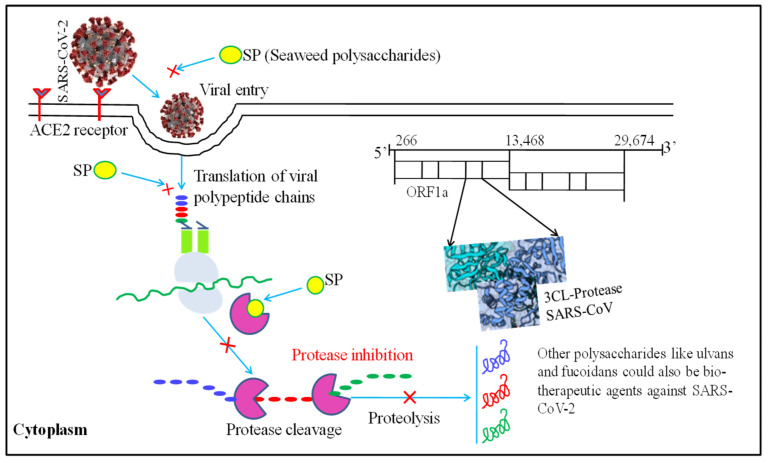
Molecular mechanism of seaweed polysaccharides (SPs) used as a potential biotherapeutic agent against SARS-CoV-2. The figure was modified from [45].

A recent review emphasized the possibility of employing SP-derived therapy to combat COVID-19 disease (Figure 2). This study was based on *Porphyridium* antiviral activity against various viruses, including HSV, varicella-zoster virus, hepatitis B virus, vaccinia virus and retroviruses. These antiviral compounds are thought to have an effect against SARS-CoV-2 [123,124,125,126]. In vitro SPs (fucoidans) derived from the macroalga *Saccharina japonica* also effectively inhibited SARS-CoV-2 [122]. RPI-27 and RPI-28 fucoidans showed more effectiveness against SARS-CoV-2 than remdesivir, an antiviral drug.

Furthermore, these fucoidans have many branches that were shown to limit the viral infection by interfering with the viral S-proteins binding to the host cells heparan sulfate co-receptor. As a result, the study revealed that fucoidans could be used alone or in conjunction with other antiviral drugs and could be a viable treatment option for SARS-CoV-2 infection [120]. These findings suggest that algal sulfated polysaccharides may have medicinal potential.

### 2.4. Algal Polyphenols as Antiviral Therapeutics against SARS-CoV-2

Polyphenols derived from brown algae, commonly known as phlorotannins, have shown promising antiviral action against viruses. Phlorotannins 8,4-dieckol and 8,8-bieckol derivatives produced from *Ecklonia cava* showed significant inhibitory results on HIV-1 reverse transcriptase and protease activities [127]. 8,4-Dieckol has also been proven to prevent syncytia formation, the synthesis of viral antigen and HIV-1 lytic effects, making it an intriguing antiviral option for future investigation [128]. Polyphenols and their derivatives were abundant in extracts from different Mexican seaweeds. Polyphenols were discovered to limit the adsorption and penetration of the Measles virus into target cells. The synergistic effects of sulfated polysaccharides and polyphenols could be an efficient source of protective and curative therapies for the measles virus that causes viral illnesses [129].

Furthermore, nine phlorotannins that were identified from *E. cava* inhibited the SARS-CoV chymotrypsin-like protease (3CL^pr^), also named as major protease (M^pro^) of SARS-CoV, which is required to reproduce the severe acute respiratory syndrome coronavirus. Eight phlorotannins were revealed to be capable of suppressing proteinase activity. According to studies on surface plasmon resonance and molecular docking, the antiviral phlorotannin dieckol was discovered to be more effective because it binds to SARS-3CL CoVs protease more effectively [130]. Dieckol was created by joining two groups, with diphenyl ether having the most effective proteinase inhibitory activity. According to a study of its kinetic mechanism, dieckol had a competitive inhibitory effect on the SARS-CoV 3CL proteinase. When phenolic chemicals produced from plants were compared to previously reported ones, dieckol was more effective at blocking proteinase cleavage. Dieckol interaction with protein residues on the SARS-CoV ligand-binding site was investigated using molecular docking. Compared to other phlorotannins, the dieckol had the lowest binding energy; it was concluded that dieckol had the highest association rate with the catalytic groups (dyad) of SARS-CoV 3CL protease [131,132]. Phloroglucinol oligomers and phlorotannins obtained from the brown alga *Sargassum spinuligerum* were discovered to be potent SARS-CoV- inhibitors. The phlorotannins 6,6-bieckol, 8,8-bieckol and dieckol produced from the marine brown alga *E. cava* were found and confirmed to be the most active and interacting protease inhibitors [132].

### 2.5. Antioxidant Potential of Algal Metabolites and Therapeutics against SARS-CoV-2

In human beings, the optimum concentration of antioxidant molecules is always required to neutralize the free radicals generated through various metabolic processes of the body or stress conditions [133]. The antioxidant diversity of algal metabolites is well documented and this metabolic diversity serves as adaptive flexibility in extreme environments to cope with environmental fluctuations and various oxidative stress-related disorders. The physiological and metabolic activities of algae that produce different metabolites showed excellent antioxidant activity. For instance, Duan et al. [134] extracted brominated mono- and bis-phenol from Symphyocladia latiuscula having free radical scavenging activity. Choi et al. [135] reported cyclohexanonyl bromophenol from the same red alga Symphyocladia latiuscula with 1,1-diphenyl-2-picrylhydrazyl showed radical (DPPH) scavenging activity.

Similarly, Li et al. [136] reported bromophenols from the marine red alga Polysiphonia urceolata, showing DPPH radical scavenging activity. In another study, Li et al. [137] reported rhodomelin A from the Rhodomela confervoides having strong radical scavenging activity. Details of some algae-derived metabolites having potent antioxidant activities are described in Table 3.

**Figure 3:**
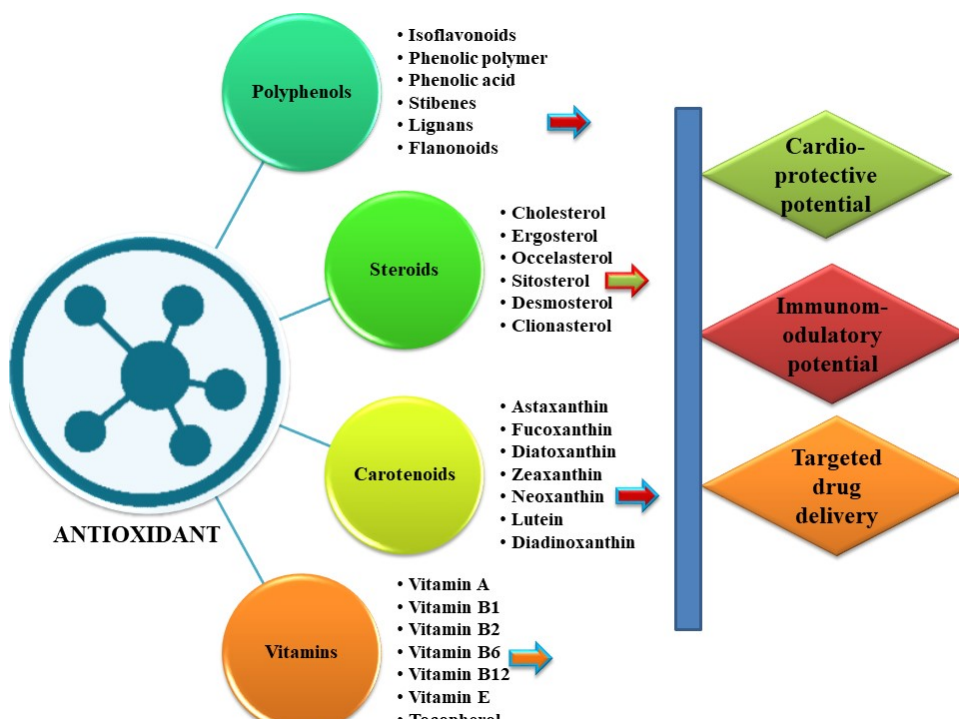
An overview of the diversity of physiologically active antioxidants produced in algae and their possible therapeutic and biological potential.

Together with antiviral medicines, antioxidants play a role in treating viral disorders by reducing oxidative stress promoting viral infections [149] (Figure 3). Influenza virus- and HIV-induced free radicals stimulated oxidative stress and reactive oxygen species (ROS) [150]. As a result, the T-cell response was triggered and total immunological protection was strengthened [151].

It is well established that natural plant products are a rich source of bioactive compounds and possess significant antioxidant activity [152]. ROS generation also plays a substantial role in SARS-CoV infection, as it causes oxidative damage, inflammation, lung infection and epithelial tissue degeneration [153,154]. After infection, SARS-CoV 3CLpro substantially enhances ROS generation in the HL-CZ cells through activating the NF-kB-dependent reporter gene, which may disrupt or imbalance the oxidation–reduction processes of the cell, resulting in oxidative stress and cellular damage. In a study, the ROS-activated NF-kB signal transduction pathway had a critical role in SARS-CoV infection [154]. Therefore, experts have recommended using antioxidants as a preventive measure to control SARS-CoV-2 infection to a certain extent [155,156,157].

## 3. Conclusions

SARS-CoV-2 is an emerging pathogen and is the cause of a pandemic outbreak around the globe. Currently, the COVID-19 problem is causing significant morbidity, mortality and socioeconomic losses. Coronavirus SARS-CoV-2 causes respiratory diseases, leading to death in extreme situations. SARS-CoV-2 has many variants already and continued viral genome mutations may lead to a rise in the number of viral variations in the future, resulting in vaccine development failure. Algal metabolites have demonstrated multistep antiviral capability, including virus binding, cell-to-cell transmission, reproduction in host cells and cytopathic effects without causing significant harm to the host cells. New research sheds information on the antiviral activities of algal metabolites, both specific and broad spectrum, particularly on drug-resistant types, indicating the necessity for more research on COVID-19 using algal metabolites. Based on the current study, algal metabolites may provide new paths for forming new therapeutics methods for treating COVID-19 and other viral diseases that are prevalent around the world. Based on published findings, we conclude that algal metabolites have remarkable potential for creating new antiviral therapies and are easily cultivable in controlled circumstances in any part of the world, regardless of geographical distribution.

## Figures and Tables

**Table 1:** List of algae-derived metabolites and their bioactivity as antiviral compounds.

Algal Species	Antiviral Metabolites	Mechanisms of Action	References
*Laminaria japonica*, *Laminaria digitata*	Alginate	Inhibition of inverse transcriptase in the RNA virus	[57]
*Gigartina skottsbergii*	Carrageenan	Inhibition of binding or internalization of viruses into host cells	[58,59]
*Ecklonia cava*	Dieckol; 8,8-bieckol	Protease inhibitor	[60]
*Porphyridium* sp.	Exopolysaccharides	Internalization or virus binding on host cells is inhibited.	[58]
*Adenocytis utricularis*, *Cystoseira indica*, *Fucus vesiculosus*, *Undaria pinnatifid*	Fucoidan	Inhibition of adhesion and blocking of reverse transcriptase	[61,62]
*Griffiths* sp.	Griffithsin	Griffithsin interacts with oligosaccharides components of spike glycoproteins of the various viruses.	[39,63]
*Agardhiella tenera*, *Schizymenia binderi*, *Callophyllis variegata*	Galactan	Blocking of virus adhesion and replication into host cells	[64]

**Table 2:** Algae-derived antiviral pharmacologically active compounds and their targeted viruses.

Marine Algal Source	Lectin Designated	Active against Viruses	References
*Griffiths* Sp.	GRFT	SARS-CoV, HCV, HIV	[99,100,101]
*Amansia multifida, Hypnea musciformis, Bryothamnion seaforthii, Solieria filiformis, Meristiella echinocarpa*	AML, HML, BSL, Sfl, MEL	HIV and influenza	[102]
*Nostoc ellipsosporum*	Cyanovirin	HIV	[103]
*Microcystis aeruginosa*	Microvirin	HIV-1	[104]
*Microcystis Viridis*	MVL	HIV-1	[105]
*Eucheuma serrai*	ESA-2	Influenza	[106]
*Halimeda renschii*	HRL40	Influenza	[107]
*Kappaphycus alvarezii*	KAA-2	Influenza	[106]
*Scytonema varium*	Scytovirin	HCV, HIV, Ebola	[108,109]

**Table 3:** Antioxidant activity of secondary metabolites synthesized from macroalgae.

Compound	Isolation Source	Assay/Activity	References
methyl-21-yl-[5′,6′-dihydro-5′-yl-{54-(4-hydroxybenzoyl)-oxy-(52-methylbutyl)}-3′-methyl-2H-pyran]-21-methyl butanoate (1), 11-[(3′,6′-dihydro-4′-methyl-2′-oxo-2H-pyran-3′-yl)methyl]-10-methylhexyl benzoate (2) and [6-ethyl-3,4-dimethyl-(tetrahydro-2′, 2′, 6′-trimethyl-2H-pyran-3′-yl)-2,5-cycloheptadiene]-1-propanoate (3)	*Turbinaria conoides*	DPPH radical scavenging activity with IC_50_ range from 0.54 to 1.1 mg mL^−1^	[138]
Fucoidan	*Undaria pinnatifida*	DPPH radical scavenging activity	[139]
methyl *N*′-(2,3,6-tibromo-4,5-dihydroxybenzyl)-γ-ureidobutyrate	*Symphyocladia latiuscula*	DPPH radical scavenging activity: IC_50_ = 27.9 µM	[140]
Sargachromanols	*Sargassum siliquastrum*	DPPH scavenging activity IC_50_ = 0.23 mM	[141]
Odonthalol and Odonthadione	*Odonthalia corymbifera*	DPPH radical scavenging activity: IC_50_ = 24.7 ± 0.0 µM	[142]
Pheophorbide A	*Enteromorpha prolifera*	The DPPH and hydroxyl radical scavenging capacities of the chloroform fraction were compared, butylated hydroxyanisole (BHA) and α-tocopherol, at concentrations ranging from 0.25 to 1.0 mg/mL.	[143]
4′-chloro-2-hydroxyaurone and 4−chloroaurone	*Spatoglossum variabile*	O_2_^−^ scavenging activity: IC_50_ = 22.2 µM	[144]
Fucoidan	*Undaria pinnatifida*	Scavenging of DPPH radicals: 9.01 ± 1.93 µg/mL	[145]
7-epi-silphiperfolan-6β-ol and silphiperfolan-7β-ol	*Laurencia dendroidea*	Scavenging of DPPH radicals; 27.5 and 30.3% at 500 µg mL^−1^, respectively	[146]
Cystoazorones A and B and cystoazorol A	*Cystoseira abies-marina*	Scavenging of DPPH radicals: 29% at 1.06 mM	[147]
3-(2,3-dibromo-4,5-dihydroxybenzyl)pyrrolidine-2,5-dione; methyl 4-(2,3-dibromo-4,5-dihydroxybenzylamino)-4-oxobutanoat;4-(2,3-dibromo-4,5-dihydroxybenzylamino)-4-oxobutanoic acid; 3-bromo-5-hydroxy-4-methoxybenzamide; and 2-(3-bromo-5-hydroxy-4-methoxyphenyl)acetamide	*Rhodomela confervoides*	These compounds showed potent scavenging activity against DPPH radicals, with IC50 values ranging from 5.22 to 23.60 μM.	[148]

## Data Availability

Data are available within the article.
